# 5′ UTR variant in the *NDP* gene leads to incorrect splicing and familial exudative vitreoretinopathy

**DOI:** 10.1186/s13023-025-03724-1

**Published:** 2025-10-31

**Authors:** Siping Liu, Ke Xiong, Xin Jiang, Lijun Tang, Leyi Chen, Yihong Li, Bei Jia

**Affiliations:** 1 Department of Obstetrics and Gynecology, Nanfang Hospital, Southern Medical University, Guangzhou, 510515 China; 2 Department of Ophthalmology, Nanfang Hospital, Southern Medical University, Guangzhou, 510515 China

**Keywords:** Familial exudative vitreoretinopathy, *NDP* gene, 5′-untranslated regions, Splicing, Whole exome sequencing

## Abstract

**Background:**

Familial exudative vitreoretinopathy (FEVR) represents a clinically and genetically diverse ophthalmic disorder marked by incomplete development of retinal blood vessels. The *NDP* gene predominantly underlies X-linked FEVR.

**Methods:**

Copy number variation sequencing, chromosomal microarray, whole exome sequencing and Sanger sequencing were performed to identify and validate the candidate variant. The functional effect of the candidate variant was further investigated in HEK293 and HeLa cells with pcMINI and pcMINI-N vectors by means of minigene splicing assay in vitro. A summary of known pathogenic variants in the 5′-untranslated regions (5′ UTR) of the *NDP* gene and their clinical characteristics was formulated.

**Results:**

Whole exome sequencing identified a novel hemizygous 5′ UTR variant (NM_000266.4: c.-167_-166delinsAAGG) in the *NDP* gene. Sanger sequencing confirmed cosegregation of this variant with FEVR in the affected family members. Minigene splicing assays demonstrated that this variant resulted in partial deletions in exon 2. Pathogenic variations in the 5′ UTR were categorized into three types: 1. indels in dipyrimidine repeats (exon 1); 2. variants in splice sites (intron 1); and 3. variants in exon 2 (5′ UTR). Among patients with variations in dipyrimidine repeats (5 out of 8), most were diagnosed with retinopathy of prematurity (ROP). Patients with splice-site variants in intron 1 (4 out of 6) were predominantly diagnosed with Norrie disease (ND), while all patients (7 out of 7) with variations in exon 2 (5′ UTR region) were diagnosed with FEVR.

**Conclusions:**

A likely pathogenic variant was identified in 5′UTR of the *NDP* gene, and validation confirmed its impact on *NDP* splicing. The present analysis results also indicate a correlation between the location of the variations in 5′UTR and disease, providing assistance in disease prognosis.

**Supplementary Information:**

The online version contains supplementary material available at 10.1186/s13023-025-03724-1.

## Background

The *NDP* gene (OMIM 300658) encodes Norrin, a secreted cysteine-knot like growth factor that regulates the growth and development of retinal vessels [1]. Norrin is a key component of the Norrin/FZD4 signaling pathway. It is secreted by Müller glial cells and activates FZD4 receptors located on vascular endothelial tip cells [2]. Precise Norrin/FZD4 signaling is critical for proper angiogenesis. Pathogenic variants in the *NDP* gene may lead to either a severe retinal phenotype associated with hearing loss (Norrie Disease, OMIM 310600) or a moderate retinal phenotype (Exudative Vitreoretinopathy, OMIM 305390)[2, 3]. Norrie disease (ND) is an X-linked recessive disorder characterized by severe early childhood blindness. Some patients with ND also exhibit additional neurological symptoms, varying degrees of developmental delays, and may develop sensorineural deafness by the second decade of life [4]. Familial exudative vitreoretinopathy (FEVR) is an X-linked inherited vitreoretinopathy characterized by anomalous retinal vascular development. The primary hallmark of the disease is a peripheral retina lacking blood vessels, which can progress to neovascularization, exudation, hemorrhage, and retinal detachment [5]. Retinopathy of prematurity (ROP) is also a cause of blindness in premature children [6]. FEVR and ROP share similar clinical phenotypes, including peripheral avascular retina, dragging of retinal vessels, abnormalities in retinal vessel branching, retinal neovascularization, and retinal detachment [7]. Untranslated region (UTR) variants mainly contribute to moderate retinopathy [2].

Previous research has revealed a correlation between the *NDP* gene and phenotype [2, 8]. The position of the variants in the NDP protein predictably determines whether the mutation results in a ‘severe’ or ‘moderate’ disease phenotype. Similar to many haploinsufficient genes, large deletions, duplications, and null variants often lead to severe phenotypes. Missense variants can result in either severe or moderate retinopathy depending on their impact on functional regions of the NDP protein. Variants affecting cysteine residues typically lead to severe phenotypes, whereas missense variants in the LRP 5/6 binding site usually cause moderate phenotypes.

To date, over 200 different variants in the *NDP* gene have been reported as disease-causing for ND, FEVR or ROP. Fewer variants in 5′UTR are less frequently reported, and there is a lack of comprehensive summaries of variants in this region. These untranslated region variants are often overlooked by conventional bioinformatics analyses. In the present study, the aim was to investigate the cause of FEVR in a Chinese family and study the disease mechanism of 5′UTR *NDP* variants.

## Methods

### Patients

**Fig. 1:**
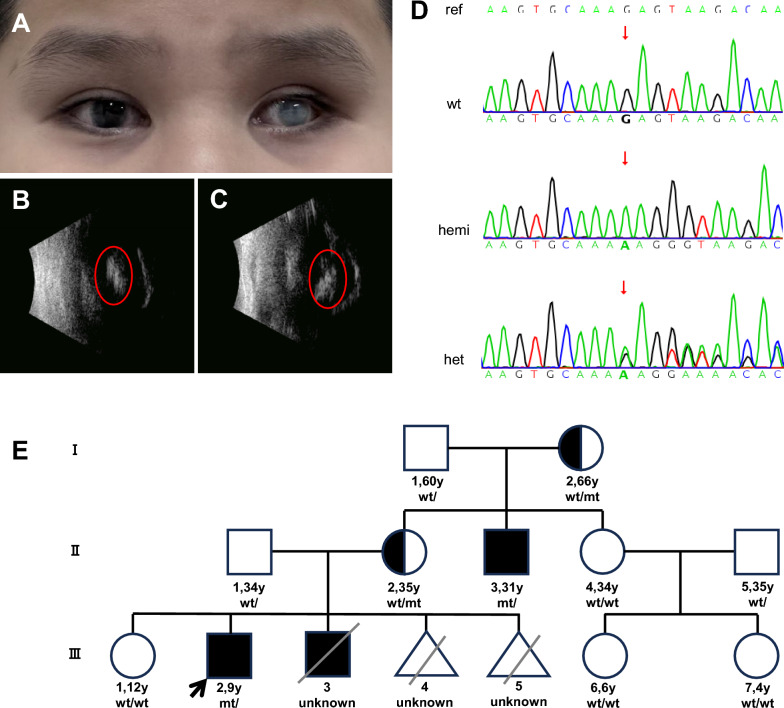
The clinical phenotype of the proband and genetic testing results **A** Binocular leukocoria in left eye; **B**, **C** Hyperechoic shadow in the middle part of the eyes; **D** Sanger sequence of NM_000266.4: c.-167_-166delinsAAGG in the *NDP* gene; **E** Pedigree of the family

The patient (III2), a 9-year-old boy, was recruited for the present study at Nanfang Hospital, Southern Medical University. He underwent pars plana vitrectomy in infancy, but with poor outcomes. His clinical symptoms included binocular vision loss, corneal degeneration, lens opacity in the right eye, and corneal leucoma in the left eye (Fig. 1A). B-ultrasound exam indicated atrophy of the eyeball and abnormal changes (Fig. 1B). Based on his clinical symptoms, he was diagnosed with FEVR. There was no blood relationship between the parents. He was the second child of his family (G5P3) with a history of FEVR. The first child (III1) was a healthy girl. The third child (III3), a boy with FEVR, passed away at one-month-old for unknown reasons. Later, the patient’s mother also experienced embryonic infertility (III4) and had an induced abortion (III5). The proband’s uncle (II3) was also diagnosed with FEVR. Genetic testing was performed when the proband was 4 years old and no pathogenic variants associated with FEVR were identified. Based on the patient’s clinical symptoms and family history, a decision was made to conduct comprehensive genetic testing of the proband and family members, including copy number variation sequencing (CNV-seq), chromosomal microarray (CMA), whole exome sequencing (WES) and Sanger sequencing.

### CNV-seq

Genomic DNA was extracted from peripheral blood samples of the proband (III2) using QIAamp® Blood Mini Kit (Qiagen, Hilden, Germany) following the manufacturer’s protocol. The quality of genomic DNA was evaluated using NanoDrop2000 and Qubit 3.0 (ThermoFisher Scientific). Subsequently, 10 ng genomic DNA was treated with NEB Next dsDNA Fragmentase (New England Biolabs, Ipswich, MA, USA) and inputted into the experimental system KR2000 (Berry Genomics, Beijing, China) to generate a PCR-free-frag library for sequencing. The sequencing of the libraries was conducted on the NextSeq CN500 platform (Berry Genomics, China) with a run time of 6.5 h. Raw reads were edited to remove artificial adaptor sequences and mapped to the GRCh37 reference genome, which was conducted using the Burrows-Wheeler Alignment (BWA) tool [9]. Reads were processed and copy number variants (CNVs) were evaluated by means of an in-house pipeline using read counts based on a smoothness model (Berry Genomics, Beijing, China)[10]. The reference databases included UCSC Genome Browser (https://genome.ucsc), Online Mendelian Inheritance in Man (http://www.omim.org/), Database of Genomic Variants (http://dgv.tcag.ca/dgv/app/home), DECIPHER, ClinVar (http://www.ncbi.nlm.nih.gov/clinvar), ISCA Database Search (https://www.iscaconsortium.org/) and ClinGen (https://www.clinicalgenome.org/). The pathogenicity of the candidate CNVs was assessed according to the guidelines outlined by the American College of Medical Genetics (ACMG) for interpretation of sequence variants [11].

### CMA

Microarray analysis using the Illumina Infinium OmniZhongHua-8 v1 BeadChip was conducted by mapping data to the GRCh37 reference genome and analyzed using KaryoStudio software as per standard protocols. Abnormal segments identified were further analyzed using UCSC Genome Browser, Online Mendelian Inheritance in Man, Database of Genomic Variants, DECIPHER, ClinVar, and ISCA Database Search. The evaluation method for CNVs aligned with CNV-seq procedures.

### WES

Peripheral blood samples were collected from the proband (III2), parents (II1, II2) and uncle (II3). Genomic DNA was extracted using the QIAamp® Blood Mini Kit (Qiagen, Hilden, Germany) and fragmented to approximately 200 bp using NEB Next dsDNA Fragmentase (New England Biolabs, Ipswich, MA, USA). The DNA fragments underwent hybridization and capture using Nano WES (Berry Genomics, China) according to the manufacturer’s protocol. Libraries were quantified using qPCR and sequenced on the Novaseq6000 platform (Illumina, San Diego, USA) in the 150 bp paired-end mode.

Raw image files were processed using CASAVA v1.82 for base calling and generating raw data. The sequencing reads were aligned to the human reference genome (GRCh38) using BWA [9], and PCR duplicates were removed using Picard v1.57 (http://picard.sourceforge.net/). The Verita Trekker® Variants Detection System by Berry Genomics and GATK (https://software.broadinstitute.org/gatk/) was employed for variant calling. Variant annotation and interpretation were conducted using ANNOVAR [12] and the Enliven® Variants Annotation Interpretation System authorized by Berry Genomics. The annotation databases mainly included OMIM (http://www.omim.org), ClinVar (http://www.ncbi.nlm.nih.gov/clinvar), HPO (https://hpo.jax.org/app/), gnomAD (http://gnomad.broadinstitute.org/) and dbSNP (http://www.ncbi.nlm.nih.gov/snp). Silico prediction algorithms included REVEL [13], SIFT (http://sift.jcvi.org), FATHMM (http://fathmm.biocompute.org.uk), MutationAssessor (http://mutationassessor.org), CADD (http://cadd.gs.washington.edu), spliceAI (https://spliceailookup.broadinstitute.org/), dbscSNV [14], MaxEntScan (http://hollywood.mit.edu/burgelab/maxent/Xmaxentscan_scoreseq.html), and RDDC (https://rddc.tsinghua-gd.org/ai/rna-splicer), among others. The candidate variants were classified into five categories “pathogenic, likely pathogenic, uncertain significance, likely benign and benign” according to the ACMG guidelines [15] and SVI General Recommendations for Using ACMG/AMP Criteria (https://www.clinicalgenome.org/working-groups/sequence-variant-interpretation/).

### Sanger sequencing

**Fig. 2:**
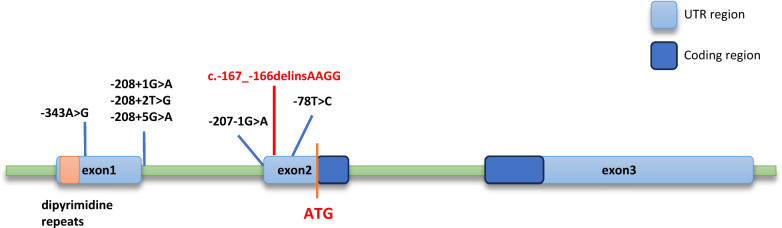
Structure of the *NDP* gene and location of the 5’UTR variations

Sanger sequencing of candidate variants in the proband and 10 family members was performed. The primers (F: GGAATGGATGACAGCCTTTG; R: ATTATCACCAGCAGGGAGAGC) were designed using Primer 5 software. PCRs were conducted under the following conditions: initial denaturation at 95 °C for 5 min, followed by 34 cycles at 95 °C for 30 s, 59 °C for 30 s and 72 °C for 15 s. PCR products were purified and sequenced using an ABI 3730XL DNA Analyzer with the BigDye™ Terminator Cycle Sequencing Kit (Applied Biosystems, Foster, CA, USA). The results of Sanger sequencing were analyzed using chromos software according to the reference sequence from GRCH38 (Fig. 2) [8, 18].

### Minigene splicing assay

**Fig. 3:**
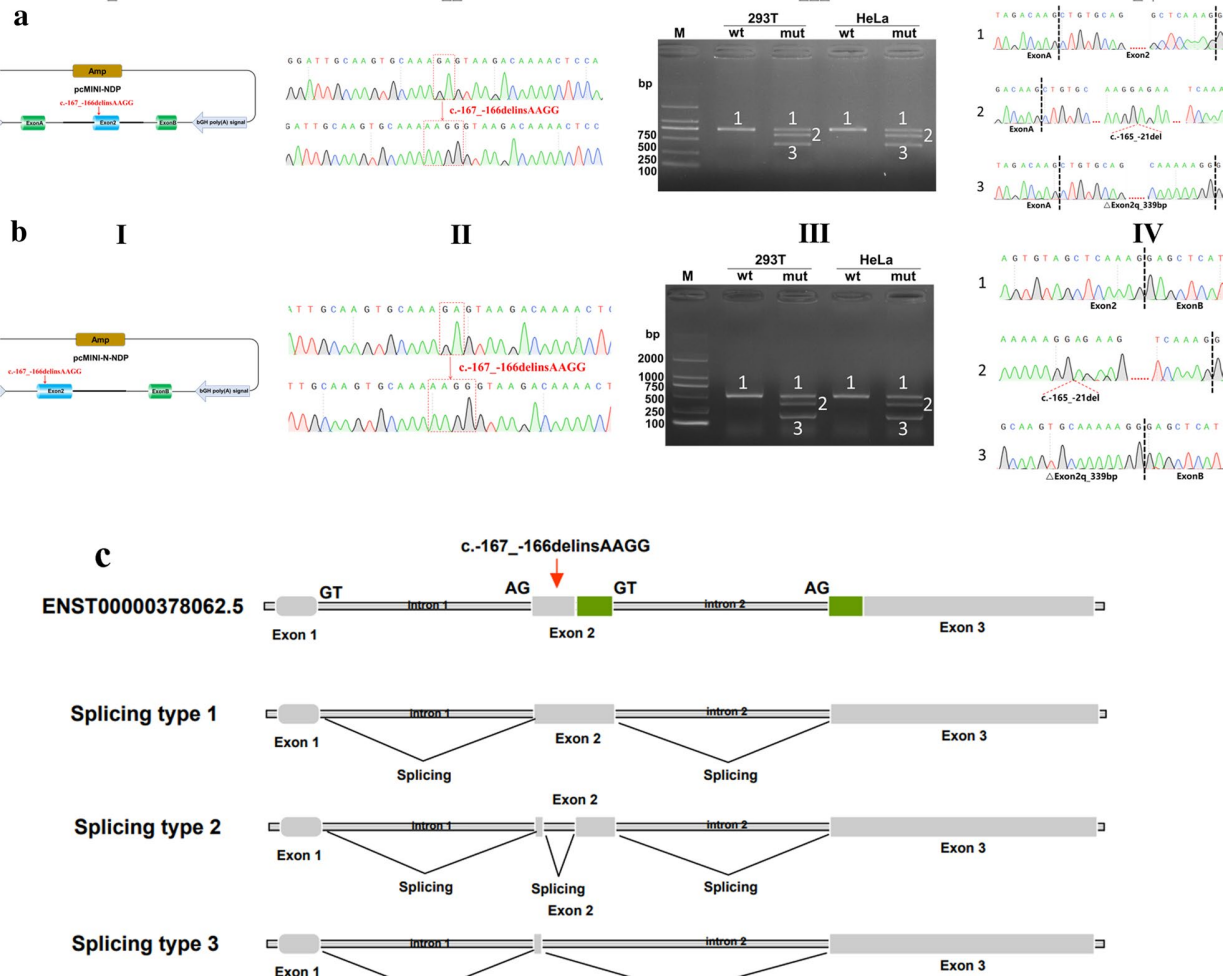
The minigene of c.-167_-166delinsAAGG in the *NDP* gene. **a**-I Structure of pcMINI vector; **a**-II Sanger sequencing of c.-167_-166delinsAAGG in pcMINI vector; **a**-III Agarose gel electrophoresis minigenes with pcMINI vector in HEK 293 T and Hela cells; **a-**IV Sanger sequencing of the products of RT-PCR; **b-**I Structure of pcMINI-N vector; **b**-II Sanger sequencing of c.-167_-166delinsAAGG in pcMINI-N vector; **b**-III Agarose gel electrophoresis minigenes with pcMINI-N vector in HEK 293 T and Hela cells; **b**-IV Sanger sequencing of the products of RT-PCR; **c** Structures of splice forms

**Fig. 4:**
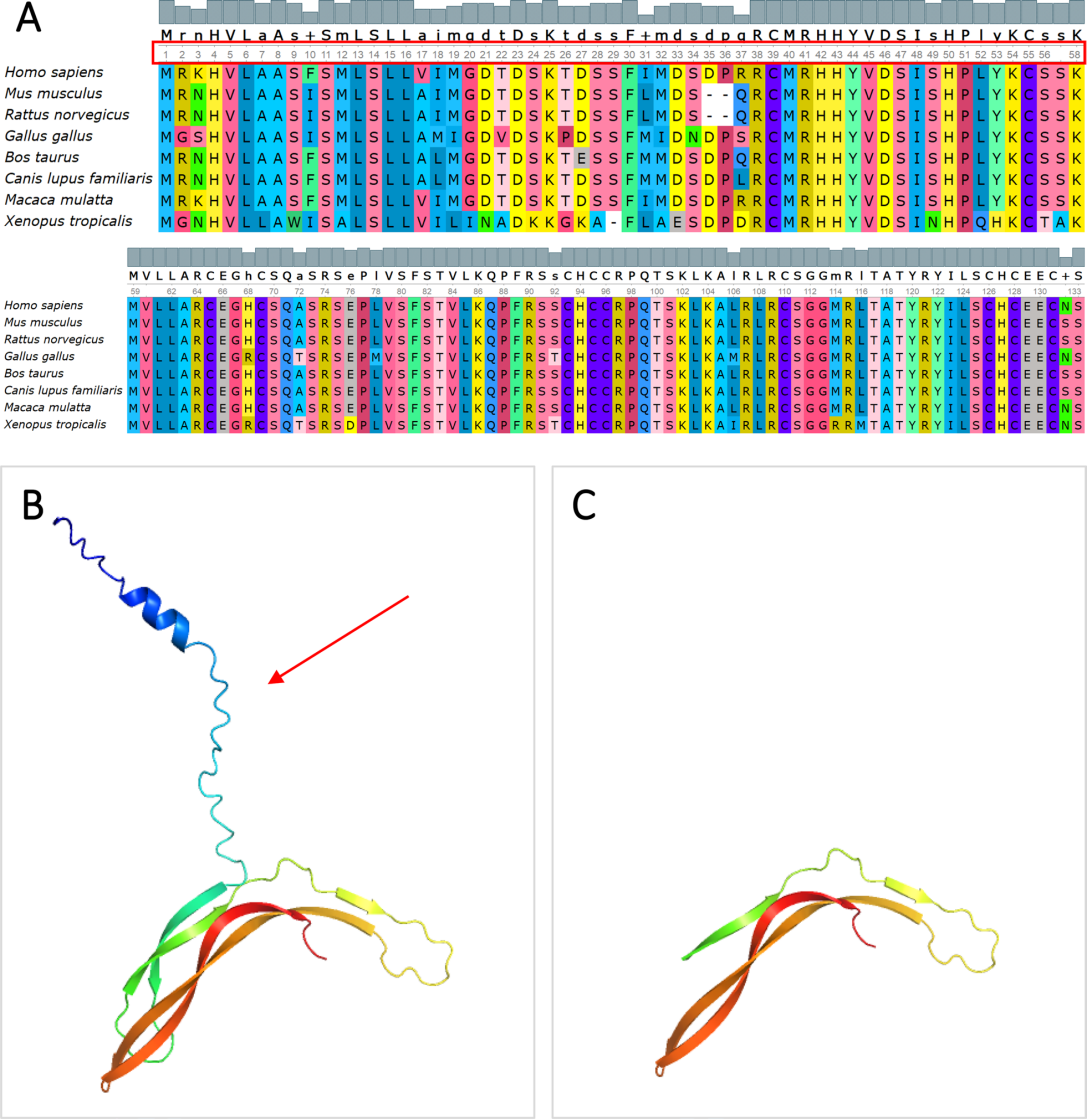
Conservatism and three-dimensional protein structure of Norrin. **A** Conservatism of Norrin; **B** Three-dimensional protein structure of Norrin; **C** Schematic of the three-dimensional protein structure of Norrin with amino acid deletion-induced conformational changes

The in vitro minigene assay was conducted to assess the impact of candidate variants on NDP gene splicing. The assay employed pcMINI and pcMINI-N vectors. Fragments of the NDP gene, including Intron1 (463 bp)-Exon2 (381 bp)-part of Intron2 (375 bp), were cloned into pcMINI plasmids (Fig. 3A), while different segments, Exon2 (381 bp)-part of Intron2 (698 bp), were inserted into pcMINI-N plasmids (Fig. 4A). Sanger sequencing confirmed wild-type (WT) and mutant pcMINI and pcMINI-N constructs. These constructs were separately transfected into HEK293T and HeLa cells using conventional restriction and ligation methods. After 48 h of incubation, total RNA was extracted from the cells using Trizol reagent (TaKaRa, Kusatsu, Japan) and reverse-transcribed with Superscript III reverse transcriptase (HifairTM 1st Strand cDNA Synthesis SuperMix for qPCR (YEASEN, Shanghai, China)). The resulting cDNA was PCR-amplified and analyzed by 1% agarose gel electrophoresis. Sequencing of the PCR products compared wild-type and mutant sequences, focusing on splice variants of the mutant type.

### Analysis of the conservative and protein structure

UGENE software (https://ugene.net/) was used to analyze the conservative of Norrin. The three-dimensional structure of Norrin was analyzed using PyMOL software (https://www.pymol.org/), with Q2KI78.1.A (https://swissmodel.expasy.org/) as the template.

### Review of pathogenic variants in 5′UTR region of the ***NDP*** gene

The variations in 5′UTR of the *NDP* gene reported in HGMD and PubMed databases were searched. Information gathered included gender, age at diagnosis, specific variation details, diagnosed disease, clinical phenotypes, and predictions from computational tools such as SpliceAI, dbscSNV, MaxEntScan, and RDDC (https://rddc.tsinghua-gd.org/ai/rna-splicer). Additionally, summaries encompassed results from functional experiments conducted on these patients.

## Results

### Analysis of CNVs

No pathogenic copy number variations were identified in both CNV-seq(seq[hg19](1–22) × 2,(XY) × 1) and CMA (arr[hg19](1–22) × 2,(XY) × 1) testing (Additional file 1).

### Sequencing of the FEVR family reveals a novel 5′UTR variant in the ***NDP*** gene

The WES and Sanger sequencing data from the family revealed a novel hemizygous variant in the NDP gene’s 5′UTR: NM_000266.4: c.-167_-166delinsAAGG. This variant was identified in two male patients diagnosed with FEVR (II3 and III2), both of whom inherited the variant from their carrier mothers (I2 and II2). Other healthy family members (I1, II1, II4, II5, III1, III6, and III7) were found to be wild type at this locus. The c.-167_-166delinsAAGG variant was originally classified as a VUS (Variant of Uncertain Significance), which is based on the following rationale: it resides within a regulatory region of incompletely understood function; there is an absence of supporting data regarding population frequency, functional experiments, or pedigree information; computational prediction results are contradictory or lack sufficient evidentiary strength; and the available data are insufficient to definitively support either a "pathogenic" or "benign" classification. Functional assays, however, provided evidence supporting its reassignment to LP (Likely Pathogenic) status. The c.-167_-166delinsAAGG variant co-segregated with FEVR within the family (PP1). It has not been reported in ClinVar or HGMD databases. Furthermore, it was absent in population frequency databases such as EXAC, gnomAD, and 1000 G (PM2_Supporting). This variant demonstrates significant enrichment in the patient population and is classified as PS4 (Moderate) based on pathogenic evidence (PS4_Moderate). The patient's phenotype demonstrates high concordance with the genetically-related disorder, warranting classification as PP4 according to diagnostic criteria (PP4). This variant occurs in exon 2, and computational predictions from RDDC and FF software suggest that it may lead to the skipping of a 339 bp segment in exon 2, including the start codon (PP3). Based on ACMG guidelines and SVI General Recommendations, c.-167_-166delinsAAGG was classified as “likely pathogenic” (PP1 + PM2_Supporting + PS4_Moderate + PP4 + PP3).

### Functional analysis of 5′UTR variant in the *NDP* gene

In the minigene splicing assay using pcMINI and pcMINI-N vectors, consistent results were observed. Upon comparing the PCR product lengths between wild-type and mutant cells, two new PCR bands (B and C) were detected, which were shorter than the wild-type band (A) (Fig. 3a-III, b-III). Sanger sequencing of band B revealed a 145 bp deletion within the middle of exon 2 (c.-167_-21delinsAAGG). Sequencing of band C showed a larger 339 bp deletion at the 3’ end of exon 2 (c.-167_174delinsAAGG), encompassing amino acids 1 to 58 (Fig. 3a-IV, b-IV, c). Amino acids 1 to 58 of Norrin are highly conserved across species (Fig. 4A). Three-dimensional protein structure diagrams illustrate the impact of the deletion of these amino acids on the protein structure (Fig. 4B, C).

### Review of variants in 5′UTR of the ***NDP*** gene

According to the data of the present study and previously reported cases, pathogenic variations in the 5′UTR were found to be distributed in three types: 1. indels located in dipyrimidine repeats within exon 1 predominantly manifest in ROP in 5 out of 8 cases, with a minority showing ND alongside symptoms like deafness and mental retardation (3 out of 8 cases); 2. variants affecting the splice region in intron 1 were associated with ND in 4 out of 6 cases, with only one instance linked to FEVR; 3. all patients (7 out of 7) harboring variations in exon 2 of the 5′UTR region were diagnosed exclusively with FEVR.

The variant c.-77A > G was observed to impact the expression levels of the *NDP* gene, whereas c.-167_-166delinsAAGG affected its splicing. Notably, functional experiments validating the pathogenicity of variations within dipyrimidine repeats and the splice region in intron 1 were not conducted. Computational predictions from multiple software tools indicate that variations in the splice region have the potential to disrupt the splicing patterns of the *NDP* gene (Table 1).

**Table 1:** Variations in 5’ UTR of *NDP* gene and clinical phenotypes

Case	Region	Variations	Sex	Age at diagnosis	disease	SpliceAI	PMID
1	exon 1	c.-386_-310del	male	6 M	ROP	N/A	11,748,312
2		c.-391_-380delCTCTCTCTCCCTinsGTCTCTC	female	34 W	ROP	N/A	11,748,312
3		c.-396_-383delTCCCTCTCTCTCTC	male	5 M	ROP	SpliceAI = G|NDP|0.00|0.00|0.00|0.00|39|-35|0|-35	17,296,899
4		ATTGTGTCCActctctctctccCCTCCAAATG(ins 12 bp)	male	1 M	ND	N/A	17,296,899
5		TCTCTCTCCCtctctctctctcccTCTCTCTCTC(del 14 bp)	male	N/A	ROP	N/A	11,322,656
6		TCTCTCTCCCtctctctctcccTCTCTCTCTC(ins 12 bp)	male	N/A	ROP	N/A	11,322,656
7		CTCTCTCTCTctctctctctCCCTCTCTCT(ins 10 bp)	male	41 Y	ND	N/A	7,627,181
8		CTCTCTCTCTctctctctctCCCTCTCTCT(ins 10 bp)	male	16 Y	ND	N/A	7,627,181
9		c.-343A > G	female	24 W	ROP	SpliceAI = C|NDP|0.00|0.00|0.00|0.00|4|-27|-26|-27	21,151,595
10	intron 1	c.-208 + 1G > A	male	9 Y	ND	SpliceAI = T|NDP|0.02|-0.00|0.00|0.99|45|-10|35|1	8,807,344
11			male	N/A	N/A		34,582,765
12		c.-208 + 2 T > G	N/A	N/A	ND	SpliceAI = C|NDP|0.04|-0.00|0.00|0.99|46|-8|34|2	20,340,138
13		c.-208 + 5G > A	male	N/A	ND	SpliceAI = T|NDP|0.00|0.00|0.00|0.13|49|-1|39|5	20,340,138
14			male	7 M	FEVR		33,588,793
15		c.-207-1G > A	male	1.5 Y	ND	SpliceAI = T|NDP|0.47|0.80|0.00|0.00|-9|-1|-9|-42	22,786,811
16	exon 2	c.-167_-166delinsAAGG	male	9 Y	FEVR	SpliceAI = CCTT|NDP|0.07|0.00|0.25|0.07|42|34|0|0	this study
17			male	31 Y	FEVR		this study
18		c.-77A > G	male	N/A	FEVR*	SpliceAI = C|NDP|0.00|0.00|0.00|0.21|-4|-2|0|-4	26,908,610
19			male	N/A	FEVR*		26,908,610
20		c.-78 T > C	male	6 M	FEVR	SpliceAI = C|NDP|0.00|0.00|0.00|0.00|-5|-3|-2|-5	33,588,793
21			male	7 Y	FEVR		33,588,793
22			male	5 Y	FEVR		33,588,793

week,W; month, M; year, Y

*patients also carry RCBTB1: c.1172 + 1G > A (p.Glu349Glyfs*17); spliceAI = ALLELE|SYMBOL|DS_AG|DS_AL|DS_DG|DS_DL|DP_AG|DP_AL|DP_DG|DP_DL

## Discussion

FEVR is a clinically and genetically heterogeneous ophthalmic disease known for its clinical and genetic diversity, characterized by incomplete development of retinal blood vessels [2, 3, 16]. In mild cases, patients may exhibit only retinal abnormalities detectable through fundus fluorescein angiography. However, severe forms of FEVR can lead to profound vision loss, with some patients registered as blind from infancy onwards. FEVR stands as a significant cause of childhood blindness, highlighting its critical impact on visual health [16, 17]. Over 11 genes have been reported as causative factors for FEVR, with the majority of these genes associated with autosomal dominant inheritance patterns of the disease [16, 17]. *NDP* is the only gene responsible for X-linked inherited FEVR, which plays a vital role in retinal angiogenesis participating in the Norrin/β-catenin signaling pathway [16]. In the present study, the characteristics of histogram exhibited typical X-linked inheritance characteristics. Therefore, a decision was made to conduct a second round of genetic testing on the family members.

The *NDP* gene contains three exons, and spans 2.8 kb. The coding region of the *NDP* gene is located in the second half of exon 2 and the first half of exon 3 [8]. The variant c.-167_-166delinsAAGG, located in the 5′ UTR of the NDP gene within the first half of exon 2, presents a challenge in verifying its pathogenicity in genetic disease diagnosis. Previous studies indicate that pathogenic variants in the 5′ UTR can potentially regulate mRNA levels or disrupt normal splicing processes, thereby impacting protein function. These mechanisms highlight the significance of investigating how such variants contribute to disease pathology, particularly in conditions like FEVR, where genetic changes can lead to diverse clinical manifestations [18].

Minigene splicing assay results verify that c.-167_-166delinsAAGG in the *NDP* gene led to two types of abnormal splicing pattern. Type 1 was a 145 bp deletion in the middle of exon 2 (c.-167_-21delinsAAGG), containing part of the 5′UTR of the *NDP* gene. Among the region of deletion, c.-77A > G and c.-78 T > C were reported in Chinese families with FEVR. Luciferase assay results of c.-77A > G indicate that the mutant led to 50% decrease in expression level [19]. Nonetheless, the pathogenic mechanism of c.-78 T > C remains unclear [8]. Type 2 refers to the 339 bp deletion at the 3′ end of exon 2 (c.-167_174delinsAAGG), encompassing both the start codon and the coding region within exon 2. This deletion aligns with predictions made by spliceAI and RDDC software. Notably, several variants affecting the start codon (c.1A > G, c.2 T > G, c.2 T > C, and c.2 T > A) have been documented as “disease-causing mutations (DM)” associated with Norrie disease [20–22]. The research of Li et al. [23] highlighted that the *NDP* gene variant c.174 + 1G > A in a Chinese family with Norrie disease resulted in a 246 bp deletion at the 3′ end of exon 2. Interestingly, both c.174 + 1G > A and c.-167_174delinsAAGG exert similar effects on the coding region of the NDP gene. This deletion potentially activated a cryptic start codon at the beginning of exon 3, leading to the production of a truncated norrin protein at its N′-end. This alteration may disrupt protein translation initiation, possibly contributing to the disease phenotype. Patients with c.174 + 1G > A typically presented with bilateral leukocoria and eyeball atrophy, without concurrent hearing loss or intellectual disability. These ocular manifestations closely resemble those observed in the proband of our study. A review encompassing twenty-five patients with *NDP* gene gross deletions or CNVs indicated that individuals with such genetic changes often exhibited severe retinal detachment either at birth or shortly thereafter. In total, 60% (3/5) of patients with deletion of exon 2 developed mental retardation [3]. Exon 2 deletions were associated with severe retinal disease, with possible neurological symptoms also of concern. Clinical management should also address the potential presence of neurological symptoms.

Previously reported pathogenic variants in 5′UTR were reviewed, and notable features were identified. The variants in 5′UTR could be divided into 3 groups: 1) indels in dipyrimidine repeats (exon1); 2) variants in the splice region (intron 1); and 3) variants in exon2 (5′UTR). The dipyrimidine repeats region located within exon 1 of the NDP gene plays a critical role in transcriptional regulation and translation efficiency. Indel variants within this region have the potential to disrupt gene regulation [24]. Historically, many indels occurring in dipyrimidine repeats have been associated with ROP, predominantly identified two decades ago using molecular marker technology. Specifically, the variant -396_-383delTCCCTCTCTCTCTC (rs770996360) has conflicting classifications in databases: “benign/likely benign” in ClinVar and “DM” (disease-causing mutation) in HGMD. However, no functional evidence currently supports these variations in dipyrimidine repeats. Further, this region is typically not covered by WES probes, and it is labeled as a low complexity region (LCR) in the gnomAD database, where NGS data reliability is reduced. No recent reports have emerged regarding new cases associated with this region.

Variants occurring in the splice donor and acceptor regions were predicted to potentially cause intron retention or exon skipping. As noted earlier, exon skipping is associated with severe retinal disease. Many patients affected by variants in the splice region (intron 1) have been diagnosed with ND, aligning with its characteristic clinical features.

In the untranslated region of exon 2, variants c.-77A > G and c.-78 T > C have been associated with FEVR. It has been documented that c.-77A > G results in a 50% reduction in *NDP* expression levels [19]. The present research validates that c.-167_-166delinsAAGG disrupts normal splicing of the *NDP* gene. Proper mRNA translation is crucial for maintaining cellular functions [25]. Through analysis of the aforementioned variations and clinical phenotypes, it was found that 5′UTR variations could lead to FEVR, ND or ROP by affecting the normal splicing and expression of mRNA. Software predictions can offer insights into abnormal splicing patterns, which can then be validated through mRNA experiments. However, accurately predicting the impact of variations in the 5′UTR region on expression levels remains challenging. In many instances, when a variation shows co-segregation within a family, it suggests a potential pathogenic role. In the present study, this family underwent multiple rounds of genetic testing, and it took five years from the initial test to identify the pathogenic variant conclusively. This highlights the complexity and difficulty involved in diagnosing pathogenic variations in the UTR region.

Most patients with FEVR caused by the *NDP* gene are male, although there have been reported cases in female patients as well. The research of Huang et al. [22] found that 33.3% (8/24) of female carriers of NDP variants exhibited typical FEVR phenotypes, while another 33.3% (8/24) showed mild vascular abnormalities. The wide spectrum of phenotypes observed in female carriers of NDP variants is presumed to be influenced by X-chromosome inactivation (XCI). XCI is a random process, and the degree of XCI can impact the severity of clinical phenotypes, as seen in conditions such as Fabry disease and Duchenne muscular dystrophy [26, 27]. Therefore, attention also needs to be paid to the ophthalmological examination and follow-up of female *NDP* heterozygous carriers.

## Conclusion

In conclusion, a likely pathogenic variant in 5′UTR of the *NDP* gene was identified in the present study. It was validated that the variant can affect the splicing of *NDP*. The present analysis results also reveal a correlation between the location of the variations in 5′UTR and disease, providing assistance in disease prognosis. However, the mechanism of pathogenic variations in the 5′UTR of the *NDP* gene still requires further research.

## Supplementary Information

Additional file 1.

Additional file 2.

## Data Availability

The data that support the findings of this study are available on request from the corresponding author. The data are not publicly available due to privacy or ethical restrictions.

## Abbreviations

5′UTR: 5′-Untranslated regions
UTR: Untranslated region
CNV-seq: Copy number variation sequencing
CMA: Chromosomal microarray
WES: Whole exome sequencing
FEVR: Familial exudative vitreoretinopathy
ND: Norrie disease
ROP: Retinopathy of prematurity
BWA: Burrows-Wheeler Alignment
CNVs: Copy number variant
ACMG: American College of Medical Genetics
LCR: Low complexity region
XCI: X-chromosome inactivation
