# Supplementary figures and images for: 5′ UTR variant in the NDP gene leads to incorrect splicing and familial exudative vitreoretinopathy
Source: Orphanet J Rare Dis. 2025 Oct 31;20:553. doi: 10.1186/s13023-025-03724-1 (PMC12577050; doi:10.1186/s13023-025-03724-1)

## Slide 1
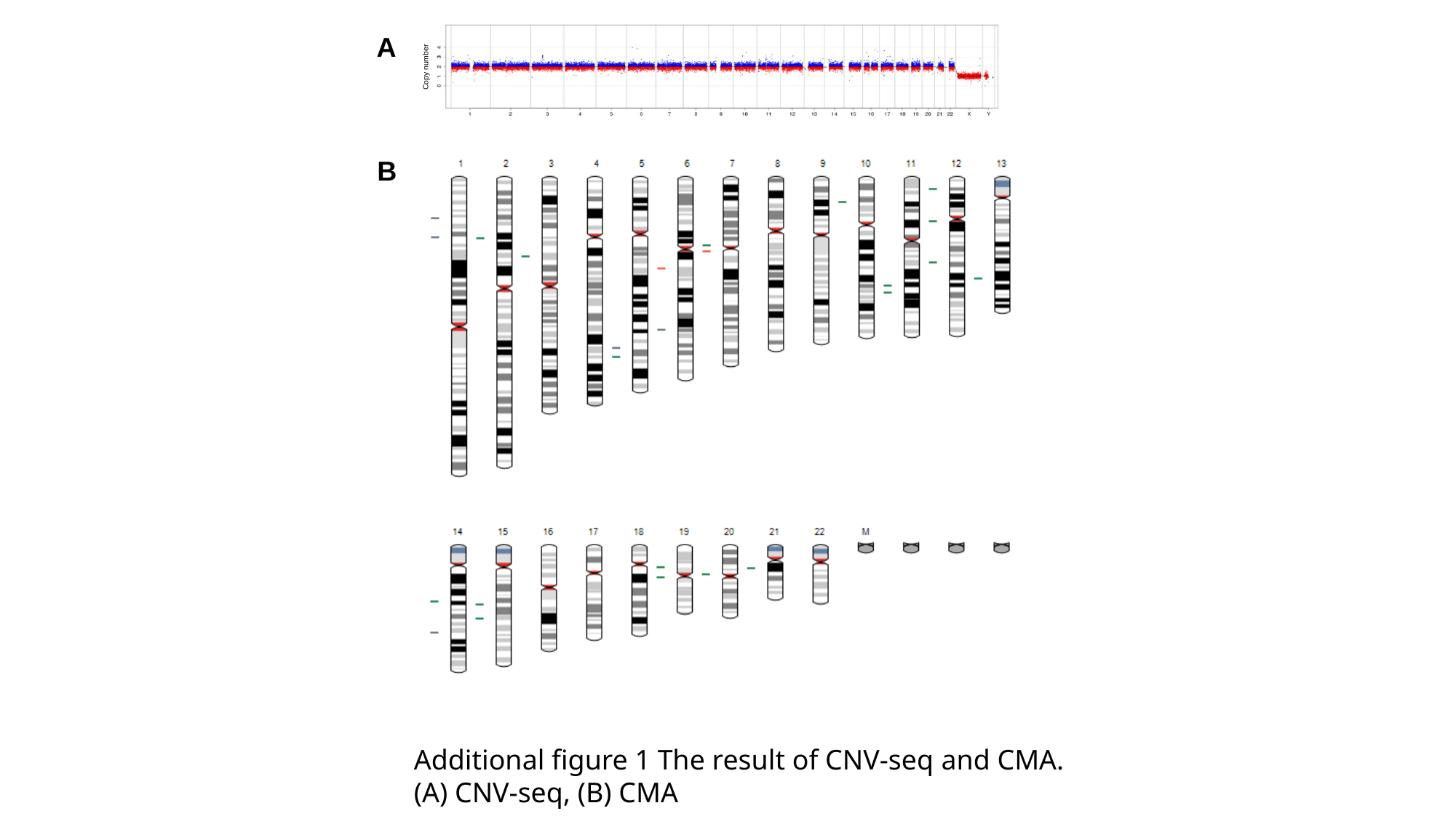

A
Copy number
B
Additional figure 1 The result of CNV-seq and CMA.
(A) CNV-seq, (B) CMA

Supplement: Supplementary file 2 — Additional file 2. [file 13023_2025_3724_MOESM2_ESM.pptx]
